# Mutational profiling can identify laryngeal dysplasia at risk of progression to invasive carcinoma

**DOI:** 10.1038/s41598-018-24780-7

**Published:** 2018-04-26

**Authors:** Lorea Manterola, Pablo Aguirre, Erika Larrea, María Arestín, Ayman Gaafar, Kepa Elorriaga, Ibai Goicoechea, María Armesto, Marta Fernández-Mercado, Ignacio Zabalza, Juan Carlos López-Duque, Ekhiñe Larruskain, Jon Alexander Sistiaga, Mikel Landa, Aitor Zabala, Francisco Santaolalla, José Antonio Municio, Ángel Ispizua, Juana María García-Pedrero, Juan Pablo Rodrigo, Charles Henderson Lawrie

**Affiliations:** 1 Molecular Oncology Group, Biodonostia Research Institute, San Sebastián, Gipuzkoa Spain; 2 Department of Pathology, Donostia University Hospital, San Sebastián, Gipuzkoa Spain; 3 Department of Pathology, Cruces University Hospital, Barakaldo, Bizkaia Spain; 4 Department of Pathology, Onkologikoa, San Sebastian, Gipuzkoa Spain; 5 Department of Pathology, Galdakao-Usansolo Hospital, Galdakao, Bizkaia Spain; 6 Department of Pathology, Basurto University Hospital, Bilbao, Bizkaia Spain; 7 Department of Otolaryngology, Donostia University Hospital, San Sebastián, Gipuzkoa Spain; 8 Department of Otolaryngology, Basurto University Hospital, Bilbao, Bizkaia Spain; 9 Department of Otolaryngology, Cruces University Hospital, Barakaldo, Bizkaia Spain; 10 Department of Otolaryngology, Hospital Universitario Central de Asturias and Instituto Universitario de Oncología del Principado de Asturias, University of Oviedo, CIBERONC, Oviedo, Asturias Spain; 11 Radcliffe Department of Medicine, University of Oxford, Oxford, UK; 12 IKERBASQUE, Basque Foundation for Science, Bilbao, Bizkaia Spain

## Abstract

Early diagnosis of laryngeal squamous cell carcinoma (LSCC) at the stage of dysplasia could greatly improve the outcome of affected patients. For the first time we compared the mutational landscape of non-progressing dysplasia (NPD; n = 42) with progressing dysplasia (PD; n = 24), along with patient-matched LSCC biopsies; a total of 90 samples. Using targeted next-generation sequencing identified non-synonymous mutations in six genes (*PIK3CA, FGFR3, TP53*, *JAK3, MET, FBXW7*), and mutations were validated by Sanger sequencing and/or qPCR. Analysis was extended *in silico* to 530 head and neck (HNSCC) cases using TCGA data. Mutations in *PIK3CA* and *FGFR3* were detected in PD and LSCC cases, as well as other HNSCC cases, but absent in NPD cases. In contrast, mutations in *JAK3*, *MET* and *FBXW7* were found in NPD cases but not PD, LSCC or other HNSCC cases. *TP53* was the most frequently mutated gene in both PD and NPD cases. With the exception of R248W, mutations were mutually exclusive. Moreover, five of seven PD mutations were located in motif H2 of p53, whereas none of the NPD mutations were. In summary, we propose that the mutational profile of laryngeal dysplasia has utility for the early detection of patients at risk of progression.

## Introduction

Head and neck cancers are the fifth most common cancer worldwide, representing over half a million newly diagnosed cases each year^1,2^. Almost 90% of these cancers originate from mucosal squamous cells and are collectively known as head and neck squamous cell carcinomas (HNSCC). Laryngeal SCC (LSCC) is the most common cancer of the larynx and the second most common respiratory cancer after lung cancer. Epidemiological studies attribute age, sex, and consumption of tobacco and alcohol, as major risk factors in the occurrence of LSCC^3^.

Despite recent advances in surgery, radiotherapy and chemotherapeutic options, the life expectancy of LSCC patients (5-year OS 50–60%) has only marginally improved over the last decades^4–6^. In particular, over half of LSCC patients with advanced disease (i.e. stage III or IV) at time of diagnosis, will have disease recurrence or distant metastatic disease, and fewer than 10–44% of these patients are cured^7^. In contrast, in early stage LSCC (i.e-stage I or II) 85% to 95% of patients can be cured through local treatment alone^8^. Consequently, there is a clear clinical need to identify new early detection biomarkers in order to develop effective prevention and treatment strategies and therefore improve the outcome for patients.

The development of LSCC is a multistep process involving structural alterations of the epithelial mucosa, from initial hyperplasia/hyperkeratosis to premalignant lesions (i.e. low/high grade dysplasia) before becoming carcinoma *in situ* (CIS), and eventually invasive carcinoma^9,10^. The percentage of laryngeal dysplasia that transform to carcinoma varies between groups; 0–11% of mild dysplasia progress to carcinoma, 4–33% of moderate dysplasia, and 10–57% of severe dysplasia^11,12^. There is, however, a lack of reliable biomarkers to predict the risk of progression from premalignant lesion to carcinoma which is compounded by the difficulty in histopathologically distinguishing between the different stages of the disease^11–13^.

It is generally accepted that progression from dysplasia to invasive malignancy is associated with cumulative genetic alterations^10^. It has been reported that LSCC arises from chromosomal alterations such as loss of heterozygosity (LOH) of 9p21, 17p13 and 18q21^14,15^. In addition, the severity of laryngeal lesions has been linked with hTERC amplification^16^, and loss of *CTNNB1* has been proposed to differ between premalignant lesions with and without progression^17^. However, none of the indicators identified to date have a reliable predictive value.

To address this issue in a comprehensive manner we used targeted next generation sequencing (NGS) approach to elucidate the mutational landscape of 24 LSCC cases along with the patient-matched antecedent laryngeal dysplasia (i.e. progressing dysplasia; PD), as well as 42 cases of laryngeal dysplasia that did not progress to carcinoma (i.e. non-progressing dysplasia, NPD). We observed a very different mutational profile between PD and NPD cases, suggesting that these mutations could be used as biomarkers for identifying patients at high risk of progression.

## Results

### Patient cohort description

The characteristics of the patient cohort used in this study largely reflect the demographics of head and neck cancer patients worldwide^18^. Patients included in this study were primarily male (59/66 (89%)), aged 36 to 89 years (median 64 years) (Table 1), and the vast majority (91%) had a history of smoking. All cases of dysplasia were located in the larynx, primarily in the glottis (47/62 (76%)), but also the subglottis and supraglottis (1/62 for each location respectively (2%)). The majority of cases were histologically defined as high-grade (46/64 (72%)) with the remainder low-grade dysplasia (18/64 (28%)). In the NPD group 57% were defined as high-grade dysplasia compared to 92% of the PD group.

**Table 1:** Summary of clinical and histopathological characteristics of samples.

	NPD	PD/LSCC	TOTAL
Patients	42	24	66
Age (years)	64 (36–89)	65 (39–86)	64 (36–89)
Gender			
Female	6	1	7
Male	36	23	59
Dysplasia			
Low-grade	16	2	18
High-grade	24	22	46
No data	2	0	2
Anatomic site			
Supraglottis	0	1	1
Glottis	38	22	60
Subglottis	1	0	1
No data	3	1	4
Follow-up (years)	7 (5–19)	5 (1–21)	6 (1–21)
Tobacco use			
Ex-smoker	4	3	7
Smoker	26	17	43
No-smoker	5	0	5
No data	7	4	11

NPD, non-progressing dysplasia; PD, progressing dysplasia; LSCC, larynx squamous cell carcinoma.

### Mutations differ between progressing and non-progressing dysplasia

Using a targeted NGS approach we interrogated >2800 cancer-associated mutation hotspots in six NPD and five PD cases along with their respective LSCC cases, and constitutional DNA. We obtained an average of 260,977 mapped reads *per* sample (96% ± 4 on target reads) and an average coverage of ~1,200 (Supplementary Table S3).

**Figure 1:**
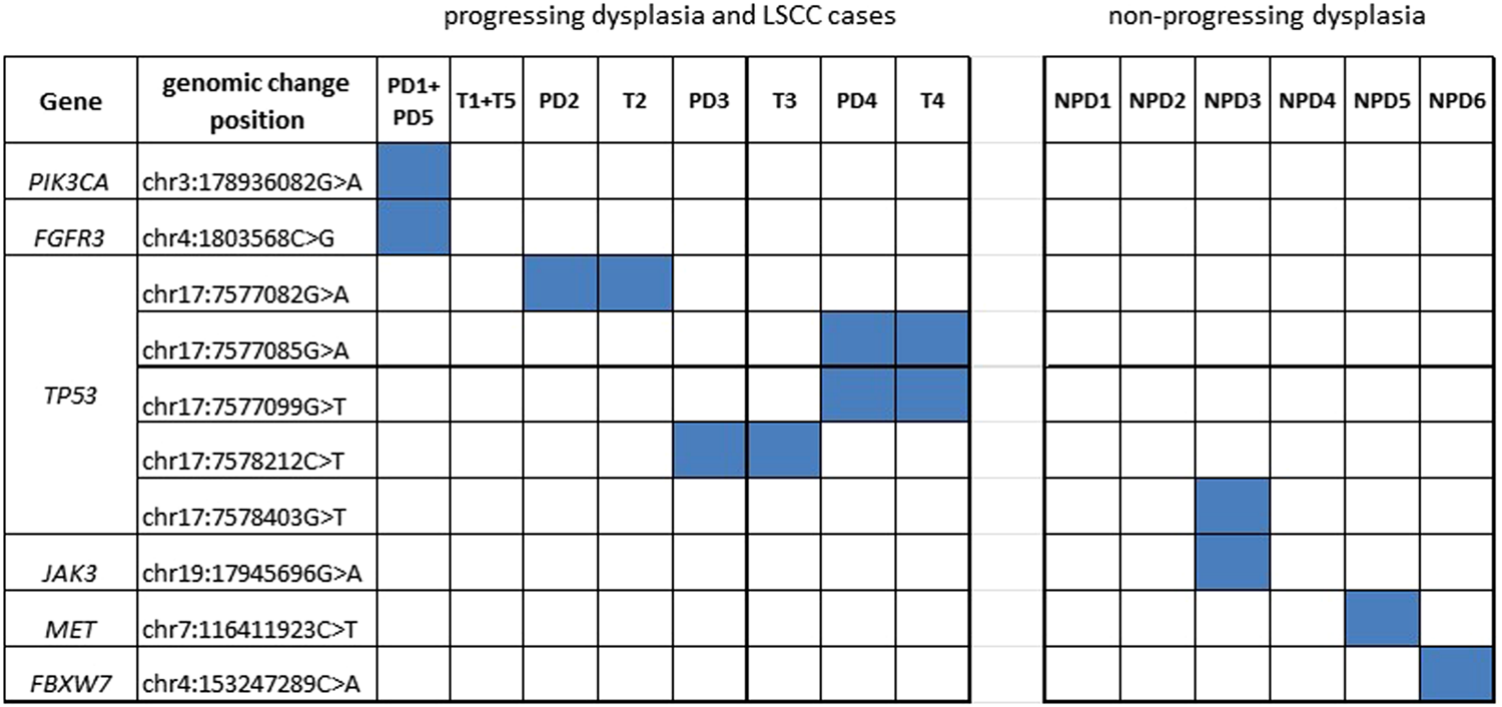
Mutations detected by NGS with a >200x coverage and >10% variant allele frequency (VAF). Mutated genes, genomic position and detected mutation in the progressing dysplasia (PD), their associated LSCC (T) and non-progressing dysplasia (NPD) are presented in blue. *The PD1 + PD5 and T1 + T5 samples were sequenced together.

Ten distinct non-synonymous mutations were detected in our samples, occurring in six different genes (*TP53*, *PIK3CA, FGFR3, JAK3, MET*, and *FBXW7*); six in PD cases and four in NPD cases (Fig. 1). The *TP53* gene was the most frequently mutated gene in 4 out of the 11 patients (36%). This gene was mutated in 3 out of 5 PD cases (60%) at four different locations. The same respective mutations were also present in the patient-matched LSCC biopsies. In contrast we only detected 1 of the 6 (16.7%) NPD cases with a mutation in the *TP53* gene. All of the PD samples harbored at least one detectable mutation, compared to half (3/6) of the NPD cases. With the exception of two cases (PD4 and NPD3) that harbored two detectable mutations, all the samples had either a single detachable mutation or no mutation at all.

Mutations were detected at six different locations in PD cases; c.1624G > A in *PIK3CA*, c.746C > G in *FGFR3* and, c.637C > T, c.839G > T, c.853G > A and c.856G > A in *TP53* (Table 2). All of these mutations are predicted to result in functional changes to the corresponding proteins, namely E542K in PIK3CA, S249C in FGFR3 and R213*, R280I, E285K, and E286K in p53. The first two of these are known activating mutations^19,20^, while the four mutations identified in *TP53*, are inactivating according to the criteria of Kato *et al.*^21^. All of these mutations are included in the COSMIC database (Catalogue of somatic mutations in cancer; http://cancer.sanger.ac.uk/cosmic)^22^. In addition, four of the mutations (i.e. E542K, S249C, R213* and R280I) are also classified as being clinically pathogenic (NCBI dbSNP database; www.ncbi.nlm.nih.gov/SNP), and all of them except R280I have previously been identified in HNSCC (Table 2).

**Table 2:** Mutations detected in PD, NPD and LSCC cases by NGS.

	Mutated sample	Gene	Genomic position	Genetic modification	AA change	Functional Classification^a^	COSMIC	dbSNP ID	MAF (ExAC)	Clinical variation^b^	References
Progressing dysplasia and associated LSCC	PD1+PD5	*PIK3CA*	chr3:178936082	c.1624G>A	p.E542K	Gain-of-function	COSM760	rs121913273	NR	Pa	^28,29,49^
	PD1+PD5	*FGFR3*	chr4:1803568	c.746C>G	p.S249C	Gain-of-function	COSM715	rs121913483	G=0.000010/1	Pa	^28,30,31^
	PD2, LSCC2	*TP53*	chr17:7577082	c.856G>A	p.E286K	Loss-of-function	COSM10726, COSM99924	NR	NA	NA	^25,29^
	PD4, LSCC4		chr17:7577085	c.853G>A	p.E285K	Loss-of-function	COSM10722; COSM137087	rs112431538	NA	NR	^30,50^
	PD4, LSCC4		chr17:7577099	c.839G>T	p.R280I	Loss-of-function	COSM11287	rs121912660	NA	Pa	
	PD3, LSCC3		chr17:7578212	c.637C>T	p.R213*	Loss-of-function	COSM10654; COSM99615, COSM99616, COSM99617, COSM99618	rs397516436	A=0.000008/1	Pa	^25,26,29^
Non-progressing dysplasia	NPD3		chr17:7578403	c.527G>T	p.C176F	Partial gain	COSM10645	NR	NA	NA	^51^
	NPD3	*JAK3*	chr19:17945696	c.2164G>A	p.V722I	Gain-of-function	COSM34213	rs3213409	T=0.0086/1048	UA	
	NPD5	*MET*	chr7:116411923	c.2962C>T	p.R988C	Gain-of-function	NOCOSMIC988	rs34589476	T=0.0029/343	US	
	NPD6	*FBXW7*	chr4:153247289	c.1273C>A	p.R425C	uncertain	NR	NR	NR	NR	

^a^The effect of the mutation on protein function was determined from published studies, and for *TP53* mutations on the overall transcriptional activity on eight different promoters as measured in yeast assays by Kato *et al.*^21^.

^b^Clinical significance on ClinVar submissions (as recommended by the American College of Medical Genetics and Genomics). MAF, minor allele frequency (1000 genomes project); PD, progressing dysplasia; LSCC, laryngeal squamous cell carcinoma; NPD, non-progressing dysplasia; chr, chromosome; NA, not applicable; NR, not reported; Pa, pathogenic; UA, untested allele; US, uncertain significance.

In contrast, from the four mutations detected in the NPD cases, only one of these, the c.527G > T mutation in *TP53* gene, a partially functional mutation^21^, has previously been reported in HNSCC (Table 2). The other three detected mutations (i.e. c.2164G > A in *JAK3*, c.2962C > T in *MET* and c.1273C > A in *FBXW7*) to the best of our knowledge have not been previously described in HNSCC. None of these mutations are classified as being clinically pathogenic by the dbSNP database, and only the *JAK3* mutation is present in the COSMIC database (in leukemias, glioblastomas and cutaneous squamous cell carcinoma) which is an activating mutation^23^.

All of the mutations detected by NGS were validated by Sanger sequencing with the exception of S249C in *FGFR3*, which we were unable to amplify despite repeated attempts (Supplementary Fig. S1).

### Validation of mutation profile in an independent cohort

We used qPCR to detect the presence of three of the mutations from the PD group (R213* and E285K in *TP53*, and E542K in *PIK3CA*), and three of the mutations from the NPD group (V722I in *JAK3*, R988C in *MET* and R425C in *FBXW7*) (Supplementary Fig. S1). Probes for the other four mutations were not available at the time. The presence of the six mutations were measured in the original discovery cohort, as well as an independent validation cohort of 36 NPD cases and 19 PD cases, along with their respective LSCC biopsies.

**Figure 2:**
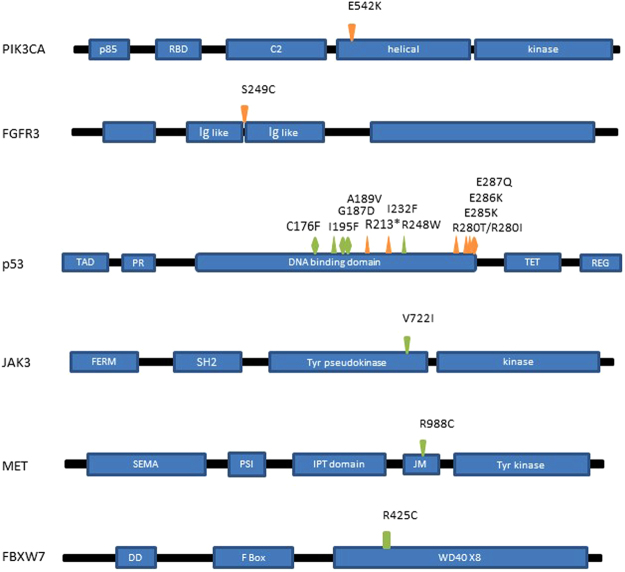
Detected mutation caused amino acid change and position in the protein. The position of the mutations are represented by arrow heads; those in orange were present in PD cases and in green NPD cases. Gain-of-function activating mutations are depicted by ▼, loss-of-function mutations by ▲, neutral mutations by ◊, and mutations with an un-known function as ▮.

In addition to validating the presence of the respective mutations in the original discovery cohort by qPCR (as well as by Sanger sequencing), we identified a further PD case (PD17) that harbored the E542K *PIK3CA* gene mutation (Fig. 2, Table 3), although in this case we did not find the corresponding mutation in the matched LSCC sample. We did not identify any further cases of the NPD-associated mutations either in NPD cases nor in the PD/LSCC cases.

**Table 3:** Mutations detected by Sanger sequencing and/or qPCR in NPD, PD and LSCC cases along with frequency of mutations in TGCA data se.

Gene	AA change	NPD cases		PD cases		LSCC cases		TCGA project			
		Mut	%	Mut	%	Mut	%	^a^LSCC Mut	%	^b^Non-LSCC HNSCC Mut	%
*PIK3CA*	E542K	0 (23)	0	2 (17)	11.7	1 (21)	4.8	3	2.6	17	4.3
*FGFR3*	S249C	0 (6)	0	1 (5)	20	0 (5)	0	0	0	4	0.9
*TP53*	E287Q	0 (13)	0	1 (16)	6.2	0 (5)	0	0	0	0	0
	E286K	0 (13)	0	1 (16)	6.2	1 (5)	20	0	0	2	0. 5
	E285K	0 (29)	0	1 (17)	5.9	1 (23)	4.3	0	0	2	0.5
	R280I	0 (13)	0	1 (16)	6.2	1 (5)	20	0	0	0	0
	R280T	0 (13)	0	1 (16)	6.2	0 (5)	0	0	0	1	0.2
	R248W	1 (13)	7.7	0 (16)	0	0 (5)	0	1	0.8	7	1.7
	I232F	0 (13)	0	1 (16)	6.2	0 (5)	0	0	0	0	0
	R213*	0 (33)	0	1 (18)	5.6	1(23)	4.3	1	0.8	11	2.7
	I195F	1 (14)	7.1	0 (16)	0	0 (5)	0	0	0	2	0.5
	A189V	1 (14)	7.1	0 (16)	0	0 (5)	0	0	0	0	0
	G187D	1 (14)	7.1	0 (16)	0	0 (5)	0	0	0	0	0
	C176F	1 (13)	7.7	0 (16)	0	0 (5)	0	0	0	1	0.2
*JAK3*	V722I	1 (22)	4.5	0 (17)	0	0 (21)	0	0	0	0	0
*MET*	R988C	1 (30)	3.3	0 (18)	0	0 (21)	0	0	0	0	0
*FBXW7*	R425C	1 (32)	3.1	0 (17)	0	0 (23)	0	0	0	0	0

^a^LSCC; larynx squamous cell carcinoma cases included in the TCGA project, n = 117.

^b^Non-LSCC HNSCC; head and neck squamous cell carcinoma that are not larynx squamous cell carcinoma cases included in the TCGA project, n = 413.

AA change, aminoacid change; NPD, non-progressing dysplasia; PD, progressing dysplasia; LSCC, progressing dysplasia associated larynx squamous cell carcinoma; TCGA, The Cancer Genome Atlas project; HNSCC, head and neck squamous cell carcinoma; Mut, mutated cases (analysed total cases); %, percentage of mutated cases relative to analysed total cases.

As mutations in the *TP53* gene have been frequently reported in HSCC, we extended our mutational profiling of this gene by sequencing exons 5 to 8, the most commonly mutated regions in HSCC. Subsequently, we identified a further three mutations (I232F, R280T and E287Q; the first two are loss-of-function and the third gain-of-function) in two of the PD samples (PD14 and PD12) and four mutations (G187D, A189V, I195F and R248W; two partial gain-of-function and two loss-of-function respectively) in four of the NPD samples (NPD26, NPD31, NPD7 and NPD11 respectively) (Fig. 2, Table 3). With the exception of E287Q, all of these mutations are listed in the COSMIC database.

### *In silico* validation

In order to extend the analysis beyond our cohorts and examine the frequency of the identified mutations in other HNSCC cases, we interrogated 117 LSCC and 413 non-LSCC HNSCC cases (530 HNSCC in total) curated in four databases via the cBioportal^24^. *TP53* harbored the most mutations in this dataset with 767 mutations in 437 cases, of which 11 cases contained the R213* mutation consisting of a single LSCC case (1/117 (<1%)) and 10 non-LSCC HNSCC cases (10/413 (3%)) (Table 3). The R248W mutation was found in 8 cases consisting of a single LSCC case (1/117 (<1%)) and 7 non-LSCC HNSCC cases (7/413 (2%)). The E286K, E285K and I195F mutations were found in two HNSCC cases (2/413 (<1%)), and neither of them were LSCC. Mutations R280T and C176F were detected in a single non-LSCC HNSCC case (1/413 (<1%)). The rest of the TP53 mutations (i.e. E287Q, R280I, I232F, A189V and G187D) were not detected in any of the samples of the TCGA project. There were 103 HNSCC samples that had mutations (n = 165) in the *PIK3CA* gene, 19% of them harboring E542K mutation (19%; 20/103); 3 LSCC cases (3%; 3/117) and 17 non-LSCC HNSCC (4%; 17/413). There were 21 *FGFR3* mutations in the TCGA samples, four of these were S249C (1%; 4/413), though all occurred in non-LSCC cases. The TCGA data set records 13 mutations in *JAK3*, 7 mutations in *MET*, and 55 mutations in the *FBXW7* gene however none of these were the same as those found in NPD cases (i.e. V722I, R988C or R425C).

## Discussion

The ability to differentiate between progressing and non-progressing dysplasia provides an important opportunity to improve survival rates, target effective treatment modalities, and improve the organ preservation rates of patients. This is the first study to look at the mutational profile by genomic techniques of a cohort of biologically homogeneous laryngeal dysplasia, and the first to note differences in the profile of dysplasia that progress to carcinoma compared to those that remain benign. Several studies have used NGS in HNSCC cases, although only a few have included LSCC cases^25–27^. It has been observed in these and other non-NGS studies that *EGFR/ERBB2* or *FGFR1/3* receptor tyrosine kinases, downstream *PIK3CA* and sometimes *HRAS* and *PTEN*, as well as tumor suppressors *CDKN2A* and *TP53* are the most frequently altered genes in HNSCC. In contrast, to date NGS has not been used to identify clinically relevant mutations associated with the risk of malignant progression to HNSCC. We therefore elucidated the mutational profile of 24 LSCC cases along with their respective antecedent PD material, in addition to carrying out the comparison with 42 NPD biopsies. For this study we interrogated >2800 cancer associated mutational hotspots in a panel of 50 cancer genes including many of the genes that are frequently mutated in HNSCC^25–31^.

Consistent with previous studies in HNSCC, we also observed that LSCC cases had frequent mutations in the *TP53* gene, as well as in *FGFR3* and *PIK3CA* genes (Fig. 1 and Table 3). Interestingly, we found that all of these mutations were also present in the antecedent dysplasia suggesting these mutations are early features in carcinogenesis and supporting the notion that they are driver mutations in carcinoma progression and mutated clone progression theory^9,10^.

In contrast, we did not detect the PD-associated mutations in our cohort of 42 NPD cases (Table 3). Furthermore, mutations associated with NPD cases (i.e. V722I, R988C and R425C) were not found in any of the 24 PD cases or their respective LSCC cases, or in the further 117 LSCC cases from database analysis, nor in the other 413 non-LSCC HNSCC cases, suggesting their presence might have utility as a biomarker of non-progression.

The most frequently mutated gene in our NGS study was *TP53* (36% of the patients; 3 out of 5 progressing and 1 out of 6 non-progressing cases). Somatic mutations in this tumour suppressor gene are common events in HNSCC, as in many other cancer types, and are found in 29–47% of HNSCC patients^25,31^. Mutations in *TP53* appear to be early events in the progression to carcinoma as they are also detected in premalignant lesions occurring more frequently in those cases with a greater histological severity^32–34^. For those reasons, we decided to extend the *TP53* analysis in the validation cohort sequencing exons 5 to 8 by Sanger. We detected similar number of mutations in both dysplasia cohorts and found mutations in PD samples were focused in exon 8 while mutations in NPD cases were in exon 5 to 7 (Fig. 2). It should be noted that due to the shortage of clinical material, we were unable to carry out loss-of-heterozygosity (LOH) analysis to assess whether or not mutations were monoallelic or not which is a frequent occurrence in HNSCC^35^.

Recent studies have aimed to classify *TP53* mutations on the basis of the change to different domains in the protein structure. For example, mutations in the DNA contact regions of the binding domain have been observed to confer a strong selection pressure to eliminate wild-type alleles in HNSCC patients and to be associated with worse prognosis^35–38^. Consistent with this idea the *TP53* mutations we found in the PD group (R280I, E285K and E286K in the original cohort and R280T and E287Q in the validation cohort) were all located in the DNA binding domain mainly affecting the helix-loop-helix motif H2. While none of the TP53 mutations detected in the NPD samples were located in that region. Although geographical variations in *TP53* mutations have been previously noted in LSCC, something that may represent a limitation in the current study, we are not aware of any evidence of this phenomena occurring in dysplasia.

PD and LSCC cases also contained mutations in the *PIK3CA* and *FGFR3* genes at hotspots that have previously been identified as oncogenic mutations, not only in LSCC and HNSCC but also in other cancer types^19,20,39,40^. Consistent with previous studies we found E542K mutation in the *PIK3CA* was frequent in PD cases (11%, 2/17)^27,41^. Interestingly, we detected the E542K mutation in both PD and the corresponding LSCC but not in NPD cases, suggesting it represents an earlier event in progression.

In contrast to the PD/LSCC-associated mutations none of the NPD-associated mutations were present in 530 HNSCC cases included in the TCGA project. Although *FBXW7* mutations have been observed in 5% of HNSCC cases^25^, none of these mutations correspond to the R425C mutation we detected. *JAK3* and *MET* genes are infrequently mutated in HNSCC (2% (12/530) and 1% (6/530), respectively). Even though the V722I *JAK3* and R988C *MET* mutations have not been detected in HNSCC cases, they have been described as activator mutations in haematological neoplasms^23,42^, as well as in several other solid tumours^43–45^. Due to sample limitation we cannot rule out that those mutations are SNPs, but as their frequency in the population is lower than 1%, according to dbSNP database, this would seem unlikely.

In summary, we demonstrate that PDs are associated with the presence of specific mutations in *TP53* and *PIK3CA* genes, whilst these mutations are absent in NPD cases and instead harbor specific mutations in *JAK3*, *MET* and *FBXW7*, that are not present in PD or other HSCC cases. We therefore propose that the mutational profile of (FFPE) biopsies from laryngeal dysplasia cases by either targeted-NGS or TaqMan-based assays could be used to distinguish between cases that remain benign, and therefore require no further clinical intervention, from those patients that are likely to progress and may benefit from up-front treatment.

## Material and Methods

### Patient description and clinical data

Formalin-fixed, paraffin embedded (FFPE) surgical tissue specimens from 66 patients diagnosed of laryngeal dysplasia between 1995 and 2011 were obtained from the Pathology Departments of six hospitals in Spain (Hospital Universitario Central de Asturias, Donostia University Hospital, Cruces University Hospital, Onkologikoa, Galdakao-Usansolo Hospital, Basurto University Hospital). This study was approved by local ethic committees (Euskadi Clinical Research Ethic Committee, approval number PI2014097). Written informed consent was obtained from patients for the inclusion of their samples in this study and samples were collected in accordance with the Declaration of Helsinki.

The clinical data of cases used in this study are described in Table 1 and Supplementary Table S1. All the cases underwent an excisional biopsy of the lesion and were re-reviewed by an experienced HNSCC pathologist (PA) to unify pathological diagnosis using morphological criteria based on WHO classification system for HNSCC^46^. NPD cases were defined as cases of dysplasia where no carcinoma was recorded to occur in a minimum of 5 years after initial biopsy (n = 42). PD cases developed invasive carcinoma at the same site between 12–60 months after initial biopsy (n = 24). Cases with diagnosis of dysplasia and invasive carcinoma in the same biopsy, or cases that developed carcinoma within 12 months of initial biopsy were excluded from the study, as were cases with a previous history of head and neck cancer or prior radiotherapy or chemotherapy treatment. Thirteen patients in the study had partial or incomplete clinical data.

### Targeted next-generation sequencing (NGS)

Targeted NGS was used to analyze FFPE tissue from 6 NPD and 5 paired PD-LSCC cases. DNA from peripheral blood mononuclear cells was used as constitutional control for patients PD2, PD3 and PD5.

DNA was purified from FFPE tissue using the QIAamp DNA FFPE tissue kit (Qiagen, Venlo, The Netherlands), and from blood cells using the Nucleospin Tissue kit (Macherey-Nagel, Düren, Germany). DNA concentration was measured using a Qubit® dsDNA HS Assay Kit (Invitrogen, Eugene, Oregon, USA) and the Nanodrop spectrophotometer (ND1000; NanoDrop Technologies, Thermo Fisher Scientific, Waltham, MA, USA).

Libraries for sequencing were constructed using the Ion Ampliseq Cancer Hotspot Panel v.2 primer pool (Thermo Fisher Scientific) as described by the manufacturer. DNA from PD1 and PD5 and their associated LSCC (LSCC1 and LSCC5) were sequenced as a pool. For each sample, 100-ng of DNA was barcoded using the Ion Xpress Barcode adapter kit and multiplexed for emulsion PCR. The resulting amplicons were sequenced using the Ion Sequencing kit v. 2.0 and an Ion 316 Chip (8 samples/chip) on an Ion Torrent Personal Genome Machine (PGM) (Thermo Fisher Scientific).

Data were analysed using Torrent Suite Software v.5.0 which was used to remove adapter sequences and align reads to the hg19 human reference genome along with the identification of variants (Variant Caller plugin v.5.0.4.0), and the Ion Reporter v.5.0 was used for mutation annotation (Thermo Fisher Scientific). We used a minimum coverage of 200 reads and >10% variant allele frequency (VAF) as a cut-off as previously described^47,48^. Additionally, variants present in the population with a minor allele frequency (MAF) greater than 1% according to the 1000 Genomes Project within the dbSNP database (https://www.ncbi.nlm.nih.gov/SNP/) were removed from analysis. Intronic and synonymous exonic mutations were also excluded. Somatic mutations were identified by comparing with their respective constitutional DNA controls.

### Mutation detection by Sanger sequencing

Selected mutations were confirmed and *TP53* exon’s 5 to 8 analysed by Sanger capillary sequencing. Seventy five ng of DNA were amplified by PCR using BioTaq DNA polymerase (Bioline GmbH, Germany), and oligos designed using Primer3 free software (http://primer3.ut.ee) (Table 2). Amplified products were resolved on 1.8% agarose gel, and purified with ExoSAP–IT (USB Corporation) or QIAquick PCR Purification Kits (Qiagen). Amplicons were sequenced with Big Dye® Terminator kit v3.1, (Thermo Fisher Scientific) on a ABI 3130xl Sequence Genetic Analyzer (Thermo Fisher Scientific) and data were analysed with Mutation Surveyor version 3.0 (SoftGenetics, State Collage, PA, USA).

### Mutation detection by quantative PCR (qPCR)

qPCR was used to validate selected mutations in an independent validation cohort that included 36 NPD, 19 paired PD and LSCC cases, 4 LSCC cases developed in NPD patients more than 6 years after dysplasia diagnosis (Supplementary Table S1) and adjacent non-tumour tissue (78 samples and 31 controls in total). Ten ng of DNA were amplified using TaqMan genotyping assays and master mix in a LightCycler 480 instrument according to the manufacturer’s instructions (Roche Diagnostics, Mannheim, Germany). Pre-designed TaqMan assay Id C_153028087, C_27862184 and C_11461286 (Thermo Fisher Scientific) were used to detect E285K mutation in *TP53*, S249C in *FGFR3* and V722I in *JAK3* respectively. Custom TaqMan assays were designed using the Custom TaqMan® Assay Design Tool (Thermo Fisher Scientific) to detect R213* mutation in *TP53*, E542K and E545K in *PIK3CA*, R988C in *MET* and R425C in *FBXW7*. The sensitivity of all the assays were tested using serial dilutions of genomic DNA (concentration range 1–100 ng) from Sanger-sequenced validated mutated and wild type samples, as well as serial dilutions of mutated DNA in wild type DNA.

### *In silico* data analysis

In order to extend the analysis, detected mutations frequency was interrogated in data from 117 LSCC cases and 413 non-LSCC HNSCC cases via cBioportal (http://www.cbioportal.org/)^24^ that includes data from Johns Hopkins^25^, the Broad Institute^26^, and the TCGA project^27^; 530 cases in total.

## Electronic supplementary material

Supplementary Tables + figure
