# Supplementary material for: Mutational profiling can identify laryngeal dysplasia at risk of progression to invasive carcinoma
Source: Sci Rep. 2018 Apr 26;8:6613. doi: 10.1038/s41598-018-24780-7 (PMC5919930; doi:10.1038/s41598-018-24780-7)
Supplement: Supplementary file 1 — Supplementary Tables + figure [file 41598_2018_24780_MOESM1_ESM.doc]

**Mutational profiling can identify laryngeal dysplasia at risk of progression to invasive carcinoma**

Lorea Manterola^a^, Pablo Aguirre^b^, Erika Larrea^a^, María Arestín^a^, Ayman Gaafar^c^, Kepa Elorriaga^d^, Ibai Goicoechea^a^, María Armesto^a^, Marta Fernández-Mercado^a^, Ignacio Zabalza^e^, Juan Carlos López-Duque^f^, Ekhiñe Larruskain^g^, Jon Alexander Sistiaga^g^, Mikel Landa^g^, Aitor Zabala^h^, Francisco Santaolalla^h^, José Antonio Municio^i^, Ángel Ispizua^g^, Juana María García-Pedrero^j^, Juan Pablo Rodrigo^j^, Charles Henderson Lawrie^a,k,l,*^.

^a^Molecular Oncology Group, Biodonostia Research Institute, San Sebastián, Gipuzkoa, Spain

^b^Department of Pathology, Donostia University Hospital, San Sebastián, Gipuzkoa, Spain

^c^Department of Pathology, Cruces University Hospital, Barakaldo, Bizkaia, Spain

^d^Department of Pathology, Onkologikoa, San Sebastian, Gipuzkoa, Spain

^e^Department of Pathology, Galdakao-Usansolo Hospital, Galdakao, Bizkaia, Spain

^f^Department of Pathology, Basurto University Hospital, Bilbao, Bizkaia, Spain

^g^Department of Otolaryngology, Donostia University Hospital, San Sebastián, Gipuzkoa, Spain

^h^Department of Otolaryngology, Basurto University Hospital, Bilbao, Bizkaia, Spain

^i^Department of Otolaryngology, Cruces University Hospital, Barakaldo, Bizkaia, Spain

^j^Department of Otolaryngology, Hospital Universitario Central de Asturias and Instituto Universitario de Oncología del Principado de Asturias, University of Oviedo, CIBERONC, Oviedo, Asturias, Spain.

^k^Radcliffe Department of Medicine, University of Oxford, Oxford, UK

^l^IKERBASQUE, Basque Foundation for Science, Bilbao, Bizkaia, Spain

^*^**Corresponding author**.

Address: Molecular Oncology group, Biodonostia Research Institute, Paseo Doctor Beguiristain s/n, 20014 Donostia-San Sebastián, Gipuzkoa, Spain.

Tel.: +34 943006138; fax: +34 943006250.

E-mail: [charles.lawrie@biodonostia.org](mailto:charles.lawrie@biodonostia.org) (CH Lawrie)

Table S1. Patient description.

| **Dysplasia** | **LSCC** | **Age at diagnosis** | **Gender** | **Dysplasia diagnosis**  **(grade)** | **Location** | **Time to carcinoma (months)** | **Follow-up (months)** | **Smoking habit** |
| --- | --- | --- | --- | --- | --- | --- | --- | --- |
| PD1 | LSCC1 | 76 | M | high | VC | 12 | 12 | smoker |
| PD2 | LSCC2 | 60 | M | high | RVC | 45 | 45 | smoker |
| PD3 | LSCC3 | 86 | M | high | RVC | 33 | 33 | ex-smoker |
| PD4 | LSCC4 | 73 | M | low | LVC | 13 | 13 | ex-smoker |
| PD5 | LSCC5 | 70 | M | high | LVC | 14 | 14 | smoker |
| PD6 | LSCC6 | 78 | M | high | ND | 26 | 134 | smoker |
| PD7 | LSCC7 | 76 | M | high | supraglottis | 33 | 73 | smoker |
| PD8 | LSCC8 | 71 | M | high | glottis | 13 | 48 | ex-smoker |
| PD9 | LSCC9 | 75 | M | high | RVC | 59 | 59 | smoker |
| PD10 | LSCC10 | 73 | M | high | VC | 50 | 50 | smoker |
| PD11 | LSCC11 | 55 | M | high | VC | 34 | 34 | smoker |
| PD12 | LSCC12 | 72 | M | high | VC | 18 | 18 | smoker |
| PD13 | LSCC13 | 62 | M | high | VC | 17 | 17 | smoker |
| PD14 | LSCC14 | 53 | M | low | VC | 42 | 42 | smoker |
| PD15 | LSCC15 | 50 | M | high | VC | 31 | 31 | smoker |
| PD16 | LSCC16 | 69 | M | high | VC | 35 | 35 | smoker |
| PD17 | LSCC17 | 47 | M | high | VC | 11 | 11 | smoker |
| PD18 | LSCC18 | 67 | F | high | LVC | 44 | 84 | smoker |
| PD19 | NA | 56 | M | high | LVC | 62 | 192 | smoker |
| PD20 | LSCC20 | 59 | M | high | LVC | 14 | 14 | ND |
| PD21 | LSCC21 | 50 | M | high | glottis | 14 | 108 | ND |
| PD22 | LSCC22 | 39 | M | high | glottis | 24 | 257 | ND |
| PD23 | LSCC23 | 72 | M | high | glottis | 57 | 116 | smoker |
| PD24 | LSCC24 | 62 | M | high | glottis | 26 | 36 | ND |
| NPD1 | NO | 69 | F | high | RVC | Never | 75 | no smoker |
| NPD2 | NO | 58 | M | high | VC | Never | 93 | smoker |
| NPD3 | NO | 68 | M | high | VC | Never | 68 | smoker |
| NPD4 | NO | 36 | M | high | VC | Never | 76 | smoker |
| NPD5 | NO | 62 | M | high | VC | Never | 72 | smoker |
| NPD6 | NO | 72 | M | high | VC | Never | 60 | smoker |
| NPD7 | NO | 77 | M | high | LVC | Never | 75 | smoker |
| NPD8 | NO | 70 | M | high | LVC | Never | 63 | no smoker |
| NPD9 | NO | 51 | M | low | LVC | Never | 55 | ex-smoker |
| NPD10 | NO | 63 | M | high | VC | Never | 95 | smoker |
| NPD11 | NO | 79 | M | high | VC | Never | 85 | smoker |
| NPD12 | NO | 89 | F | high | LVC | Never | 90 | no smoker |
| NPD13 | NO | 59 | M | high | LVC | Never | 60 | smoker |
| NPD14 | NO | 77 | M | high | both VC | Never | 65 | ex-smoker |
| NPD15 | NO | 50 | F | low | RVC | Never | 50 | smoker |
| NPD16 | NO | 62 | M | high | LVC | Never | 45 | no smoker |
| NPD17 | NO | 69 | M | high | VC | Never | 60 | smoker |
| NPD18 | NO | 43 | M | low | VC | Never | 100 | smoker |
| NPD19 | NO | 58 | M | low | VC | Never | 123 | smoker |
| NPD20 | NO | 85 | M | high | both VC | Never | 168 | smoker |
| NPD21 | NO | 77 | F | high | both VC | Never | 60 | smoker |
| NPD22 | NO | 70 | M | low | glottis | Never | 48 | smoker |
| NPD23 | NO | 61 | M | high | glottis | Never | 96 | ex-smoker |
| NPD24 | NO | 53 | M | low | LVC | Never | 72 | ND |
| NPD25 | NO | 44 | M | high | LVC | Never | 84 | smoker |
| NPD26 | NO | 70 | M | high | LVC | Never | 72 | ND |
| NPD27 | NO | 46 | M | low | glottis | Never | 96 | smoker |
| NPD28 | NO | 62 | M | low | glottis | Never | 84 | smoker |
| NPD29 | NO | 65 | M | low | glottis | Never | 108 | ND |
| NPD30 | NO | 59 | M | high | glottis | Never | 60 | smoker |
| NPD31 | NO | 73 | M | ND | LVC | Never | 72 | ND |
| NPD32 | NO | 48 | F | low | LVC | Never | 96 | smoker |
| NPD33 | NO | 73 | M | low | RVC | Never | 108 | no smoker |
| NPD34 | NO | 58 | M | low | subglottis | Never | 72 | smoker |
| NPD35 | NO | 74 | M | low | ND | Never | 72 | ND |
| NPD36 | NO | 67 | M | low | LVC | Never | 72 | ND |
| NPD37 | NO | 56 | F | low | glottis | Never | 60 | smoker |
| NPD38 | NO | 57 | M | high | LVC | Never | 60 | smoker |

PD, progressing dysplasia; NPD, non-progressing dysplasia; LSCC, larynx squamous cell carcinoma; M, male; F, female; VC, vocal cord; LVC, left vocal cord; RVC, right vocal cord; ND, no available data; NA, not available

Table S2. Primers used for mutation validation PCR (5’ → 3’)

| **Gene** | **Mutation** | **Oligo name** | **sequence** |
| --- | --- | --- | --- |
| *PIK3CA* | E542K | PIK3CA-Fw  PIK3CA-Rv | GACAAAGAACAGCTCAAAGCAA  ACATGCTGAGATCAGCCAAAT |
| *FGFR3* | S249C | FGFR3-Fw  FGFR3-Rv | GAACAAGTTTGGCAGCATCC  TGCGTCACTGTACACCTTGC |
| *TP53* | R280I/R280T, E285K, E286K, E287Q | Ex8-Fw  Ex8-Rv | AATGGGACAGGTAGGACCTG  ACCGCTTCTTGTCCTGCTTG |
|  | I232F, R248W | Ex7-Fw  Ex7-Rv | GGCCTCATCTTGGGCCTGTG  GTGTGCAGGGTGGCAAGTGG |
|  | G187D, A189V, I195F, R213* | Ex6-Fw  Ex6-Rv | AGGCCTCTGATTCCTCACTG  AGAGACCCCAGTTGCAAACC |
|  | C176F | Ex5-Fw  Ex5-Rv | TCAACTCTGTCTCCTTCCTC  CTGTGACTGCTTGTAGATGG |
| *JAK3* | V722I | JAK3-Fw  JAK3-Rv | ATAGACCCACCCCAATCTCC  TCCCACTTTCATTCCCTCAG |
| *MET* | R988C | MET-Fw  MET-Rv | TCGATTCTTGTGTGCTGTCT  CGGGCACTTACAAGCCTATC |
| *FBXW7* | R425C | FBXW7-Fw  FBXW7-Rv | ACAACCCTCCTGCCATCATA  TCGAGATGCCACTCTTAGGG |

Table S3. Targeted NGS run results.

|  |  |  |  |  |  |  | **Coverage Analysis (v5.0.4.0)** | **Variant Caller (v5.0.4.0)** |
| --- | --- | --- | --- | --- | --- | --- | --- | --- |
| **Sample** | **Mapped reads** | **On target (%)** | **Mean depth** | **Uniformity (%)** | **Amplicon read 500x (%)** | **Target base coverage at 500x (%)** | **Variants** | **Hot Spots** |
| PBMC5 | 369130 | 99 | 1716 | 99 | 99 | 99 | 10 | 2 |
| PD1+PD5 | 453769 | 96 | 1942 | 99 | 98 | 98 | 18 | 6 |
| LSCC1+LSCC5 | 339244 | 98 | 1507 | 99 | 95 | 94 | 16 | 4 |
| PBMC2 | 158384 | 99 | 734 | 99 | 77 | 75 | 13 | 2 |
| PD2 | 444685 | 99 | 1998 | 90 | 88 | 86 | 39 | 6 |
| LSCC2 | 292109 | 98 | 1298 | 99 | 90 | 88 | 16 | 4 |
| PD3 | 344552 | 98 | 1508 | 98 | 95 | 94 | 30 | 8 |
| LSCC3 | 410720 | 98 | 1792 | 97 | 96 | 95 | 50 | 13 |
| PBMC4 | 146215 | 99 | 664 | 99 | 69 | 61 | 15 | 2 |
| PD4 | 335688 | 99 | 1452 | 85 | 79 | 76 | 198 | 26 |
| LSCC4 | 404545 | 98 | 1751 | 89 | 88 | 86 | 112 | 21 |
| NPD1 | 124843 | 97 | 539 | 89 | 48 | 43 | 165 | 16 |
| NPD2 | 144114 | 92 | 594 | 99 | 64 | 58 | 29 | 9 |
| NPD3 | 121948 | 85 | 459 | 98 | 47 | 38 | 49 | 9 |
| NPD4 | 118784 | 93 | 486 | 99 | 45 | 37 | 153 | 8 |
| NPD5 | 122242 | 91 | 504 | 98 | 53 | 48 | 21 | 4 |
| NPD6 | 382254 | 98 | 1661 | 98 | 94 | 92 | 12 | 2 |
|  |  |  |  |  |  |  |  |  |
| **MEAN** | 260977 | 96 | 1131 | 95 | 73 | 70 | 68 | 10 |
| **SD** | 132030 | 4 | 595 | 5 | 20 | 22 | 65 | 7 |

c.853G>A

WT


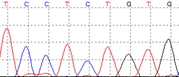

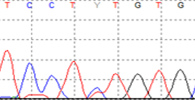


A

WT


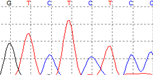

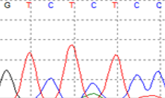


c.839G>T

B

C


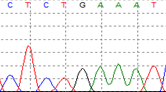


WT


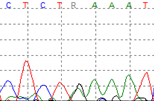


c.1624G>A


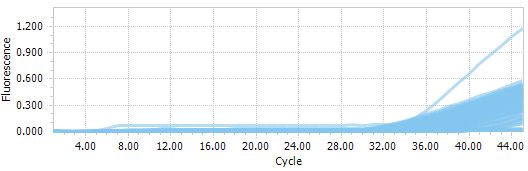


c.2164G>A in *JAK3*

NPD3

WT


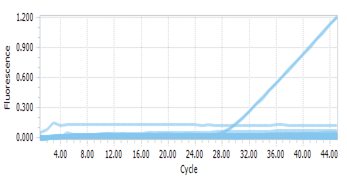


c.1273C>A in *FBXW7*

NPD6

WT


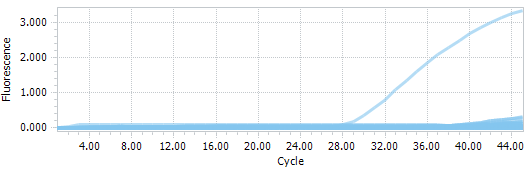


c.2962C>T in *MET*

NPD5

WT

D

E

F

**Supplementary Figure S1.** Examples of mutations confirmed by Sanger sequencing (A, B and C) and by qPCR (D, E and F). The electropherograms show mutation at codon 285 and 280 of *TP53* in sample PD4 (A and B respectively) and 542 of *PIK3CA* in sample PD1 (C ) (Table2). Fluorescence curves show mutation detection at codon 722 iof JAK3 in NPD3, 988 of MET in NPD5 and 425 of FBXW7 (D, E and F, Table2). WT, wild type sequence. Arrow heads point to the wild type and mutated allele.
